# Evaluation of a community pharmacy-led test-and-treat service for women with uncomplicated lower urinary tract infection in England

**DOI:** 10.1093/jacamr/dlaa010

**Published:** 2020-03-12

**Authors:** Tracey Thornley, Charlotte L Kirkdale, Elizabeth Beech, Philip Howard, Peter Wilson

**Affiliations:** 1 University of Nottingham, Nottingham NG7 2RD, UK; 2 Boots UK Ltd, Thane Road, Nottingham NG90 1BS, UK; 3 NHS England and NHS Improvement, Wellington House, Waterloo Road, London SE1 8UG, UK; 4 University of Leeds, Leeds LS2 9JT, UK; 5 Department of Microbiology & Virology, University College London Hospitals, London W1T 4EU, UK

## Abstract

**Background:**

Uncomplicated lower urinary tract infections (UTIs) are common in women consulting primary healthcare, taking up GP resources. Delayed consultation can increase the risk of serious infections such as pyelonephritis or bacteraemia.

**Objectives:**

To evaluate the effectiveness and uptake of a lower UTI test-and-treat service for women presenting with urinary symptoms within a community pharmacy in supporting self-care and appropriate use of antibiotics and reducing demand on other NHS resources.

**Methods:**

The service was aligned to national guidelines to diagnose and treat lower UTI in women aged 16–64 years and used national resources to provide safety-netting and self-care advice. Consultation included clinical assessment and a urine dipstick test alongside a novel smartphone app, with diagnosis informed by test results. Women were provided with safety-netting advice and either advised on self-care, supplied with antibiotics or referred to their GP.

**Results:**

Data were analysed for 764 women who presented to 23 pharmacies during December 2018 to April 2019. Lower UTI was found to be likely in 372/496 (75.0%) women, most of whom purchased antibiotics on the same day. Had the service not been available, approximately three-quarters of women who had completed the service and responded to the question would have visited their GP (214/301) and more than one-third would have used self-care with or without going to see their GP (116/301).

**Conclusions:**

A community pharmacy-led UTI test-and-treat service for women aged 16–64 years presenting with urinary symptoms provided accessible and timely care aligned to national guidance, with 75.0% of consultations requiring antibiotic treatment.

## Introduction

Urinary tract infections (UTIs) are experienced by 10%–15% of women each year^1–3^ and are the second most common condition to be prescribed antibiotics in English primary care.^4^ A survey of 2424 women found that 95% who had a UTI consulted a health professional for their symptoms and nearly two-thirds reported visiting their local GP during routine consulting hours; in addition, 14% contacted an out-of-hours service.^3^

Antimicrobial resistance (AMR) is a global problem and the UK Review on Antimicrobial Resistance has recommended all antibiotic prescriptions should be informed by data and testing technology to reduce inappropriate use.^5^ PHE has published guidance to enable the diagnosis of lower UTI in women aged <65 years who present with urinary symptoms (herein referred to as ‘PHE UTI guidance’) and defines three key urinary symptoms: dysuria, new nocturia and cloudy urine (herein referred to as ‘PHE three key symptoms’).^6^ A decision to treat with antibiotics can be made in women with two or more of these symptoms, while the additional use of a urine dipstick test may reduce diagnostic uncertainty for women who have one of these symptoms; however, up to 24% of women with a negative urine dipstick test result may have a UTI, due to issues around poor urine sample collection and the limited negative predictive values of dipstick urinalysis.^7^ The UK’s Five-Year National Action Plan aims to halve healthcare-associated Gram-negative bloodstream infections by 2024^8^ and NHS England improvement schemes have focused on reducing the risk of *Escherichia coli* bloodstream infections associated with the management of UTI in the community.^9^

Rapid diagnosis and effective early management can help reduce serious urinary infections.^10^ An analysis of 1688 patients with *E. coli* bacteraemia identified treatment for UTIs in the preceding month being a major risk for bacteraemia due to non-susceptibility of the pathogen. Close monitoring and effective treatment of patients was recommended. In another study of bacteraemia, amoxicillin/clavulanic acid-resistant infections increased in areas where use of the antibiotic was highest.^11^ Others have found little risk of pyelonephritis in those not receiving antibiotics.^12–14^ However, early appropriate treatment of symptomatic patients aged <65 years would limit the risk of inappropriate antibiotics being used.

Community pharmacy teams across the UK deliver a variety of services to support antimicrobial stewardship, including vaccination services and support for self-care. In addition, some community pharmacy schemes have been developed to provide pharmacy-led services to diagnose infections including group A streptococcal infection and chlamydia.^15^^,^^16^ Accessible opening times, convenient locations and no requirement for appointments mean community pharmacies are well placed to support patients with minor infections. The Community Pharmacy Contractual Framework 2019–2024 includes details of a new NHS Community Pharmacist Consultation Service to support urgent care referrals, many of which are for minor infections, to community pharmacy as the first port of call.^17^

There are pockets of community pharmacies across the UK that are commissioned by the NHS to provide services for managing UTIs; however, these vary greatly in eligibility criteria and method of diagnosis (to test or not to test).^18^ Published studies of community pharmacy-led UTI services report the use of clinical assessment without the additional use of a urine dipstick test.^19–21^

The objective of this study was to evaluate a private pay, test and treat service for women aged 16–64 years presenting with urinary symptoms to community pharmacies, with a subsequent diagnosis of uncomplicated lower UTI. The pilot tested the feasibility of delivering a pharmacy-led service, with the aim of understanding the effectiveness of the service in supporting self-care, appropriate use of antibiotics and reducing demand for other NHS resources.

## Methods

The service was developed by a project team and an external expert advisory panel, which included pharmacists, doctors and microbiologists. Women aged 16–64 years presenting to the community pharmacy with urinary symptoms were eligible to participate in the pilot service. They were clinically assessed for the possibility of uncomplicated lower UTI, in line with national diagnostic guidance, with treatment based on the outcome of the urine dipstick test and NICE guidance NG109.^6^^,^^22^ The patient pathway included advice on self-care, supply of antibiotics or referral to the GP, as appropriate.

Pharmacists providing the service were trained using an e-learning module, which included a clinical guide, practice scenarios and an online test, and were encouraged to sign up as Antibiotic Guardians.^23^ Pharmacy teams were provided with training material about the management of UTIs, service protocol and a link to a short training video. Pharmacists engaged with local GP surgeries to make them aware of the service. Women found out about the service through discussions with pharmacy staff, posters and leaflets displayed within the pharmacy.

Patient access to a smartphone able to use the DIP UTI test kit app was required for the urine dipstick test (Healthy.io Ltd, Tel Aviv, Israel; nitrite: sensitivity 99.3%, specificity 97.8%; blood: sensitivity 94.1%, specificity 95.3%; leucocytes: sensitivity 88.1%, specificity 95.2%). Exclusion criteria included pregnancy, diabetes, kidney problems, fever, flu-like symptoms, back pain or tenderness, nausea or vomiting, or vaginal discharge. Women were also excluded if any of the following applied: had symptoms for more than 7 days; were currently taking an antibiotic or had taken one within the previous 4 weeks for a UTI; had two or more episodes of UTI in the last 6 months or three or more in the last 12 months; used a urinary catheter; or were immunocompromised. Because patients with urinary catheters and patients aged >64 years were excluded, and because patients had to walk into a community pharmacy to access the service, it is unlikely that cases would be hospital acquired.

A pharmacy advisor explained the service to the patient, recorded their details, assessed them against the exclusion criteria and recorded the patient-reported symptoms (burning pain when passing urine, passing urine more often than usual at night, cloudy urine, blood in urine, needing to pass urine more often than usual, sudden need to pass urine, pain or discomfort in lower tummy). Eligible women who wanted to continue with the service were then required to purchase the DIP UTI Self-Testing Kit (£9.99) and download the free app. The app gave instructions to collect and analyse a mid-stream urine sample in a convenient location and stored the test result on the individual’s smartphone to share on return to the same pharmacy within 72 h. During the subsequent consultation, the pharmacist then advised on the likelihood of a lower UTI (‘UTI likely’ or ‘UTI less likely’). Treatment decisions were made on the outcome of the urine test result (lower ‘UTI likely’ if sample positive for nitrite or for leucocytes and blood).^6^ Women who were determined to be likely to have a UTI were offered antibiotic treatment at an additional cost of £14.99. First-line treatment was nitrofurantoin 100 mg prolonged-release capsules (one capsule twice daily for 3 days), with nitrofurantoin 50 mg instant-release tablets (one tablet four times a day for 3 days) where the formulation was unsuitable (e.g. vegetarian option preferable). Where nitrofurantoin was clinically unsuitable, the second-line option was trimethoprim 200 mg tablets (one tablet twice a day for 3 days). Treatment was supplied by the pharmacist under the authority of a Patient Group Direction (PGD) and was in line with NICE NG109 guidance.^22^ Pharmacists informed the women’s GP by letter of the treatment within 24 h (where consent to do so was given).

All women were provided with safety-netting (e.g. expectation around duration of symptoms; to see GP urgently if their symptoms worsened or no improvement within 48 h) and self-care advice (e.g. symptom control, future prevention advice) and a TARGET UTI leaflet.^24^ Individuals were excluded from the service and referred to a doctor if they were pregnant or had a recurrent UTI, symptoms for more than 28 days, recent UTI treated with an antibiotic, urinary catheter or any of the following: visible haematuria as the only symptom, symptoms suggestive of a sexually transmitted infection, renal impairment or diabetes. An urgent referral was made if the individual was immunocompromised, had a fever or was systemically unwell or had symptoms of upper UTI or pyelonephritis. Women were referred urgently to accident and emergency if sepsis was suspected.

Data were collected through Boots UK pharmacies (a member of Walgreens Boots Alliance, with headquarters in Nottingham). Data analysis utilized Microsoft Excel 2013. The Carstairs index was used to calculate deprivation quintiles (based on postal sector) for the least and most deprived and is based on four census indicators (low deprivation indicated by a negative value, high deprivation by a positive value).^25^

### Ethics

Anonymized copies of the data record were sent to the Boots UK head office for electronic input. The proposed work was reviewed by the Boots Research Governance group and deemed to be a service evaluation and therefore in line with NHS Health Research Authority guidelines;^26^ ethics approval was not required.

## Results

Data were collected for all women who used the service over a test period of 4 months (11 December 2018 to 11 April 2019) across 23 participating pharmacies (21 in London, 2 in Sheffield). Incomplete or illegible data were corrected by contacting participating pharmacists to clarify data entry. Where this was not possible and the data in question were of clinical importance to the analysis, data were excluded (*n *=* *28). In total, 764 complete records were returned for analysis (724 from London, 40 from Sheffield), representing 496/743 (66.8%) tests conducted during that time period among all eligible women participating in the service and 360/379 (95.0%) antibiotics supplied (compared with transactional data recorded).

**Figure 1.**
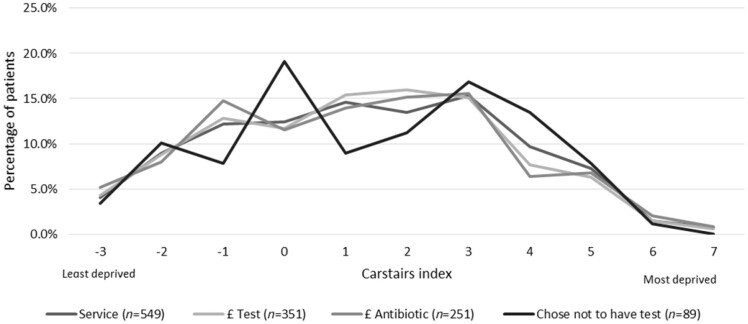
Deprivation profile for paid and non-paid elements of the service.

Data on age were available for 745 women (unknown: *n *=* *19). The mean age was 32 years and the median age was 29 years (positively skewed distribution). Most women were aged between 16 and 35 years (71.3%, 531/745). Deprivation index was calculable for 549 women and was normally distributed across the Carstairs index (Figure 1). There appeared to be little difference in the Carstairs index for those accessing the free components of the service (initial upfront advice and consultation, mean 1.3) and those that then went on to access the paid elements (test and/or antibiotics, mean 1.2). Carstairs index was calculable for 89/121 (73.6%) women who chose not to continue with purchasing the test despite being eligible, from which the mean was 1.4.

**Figure 2.**
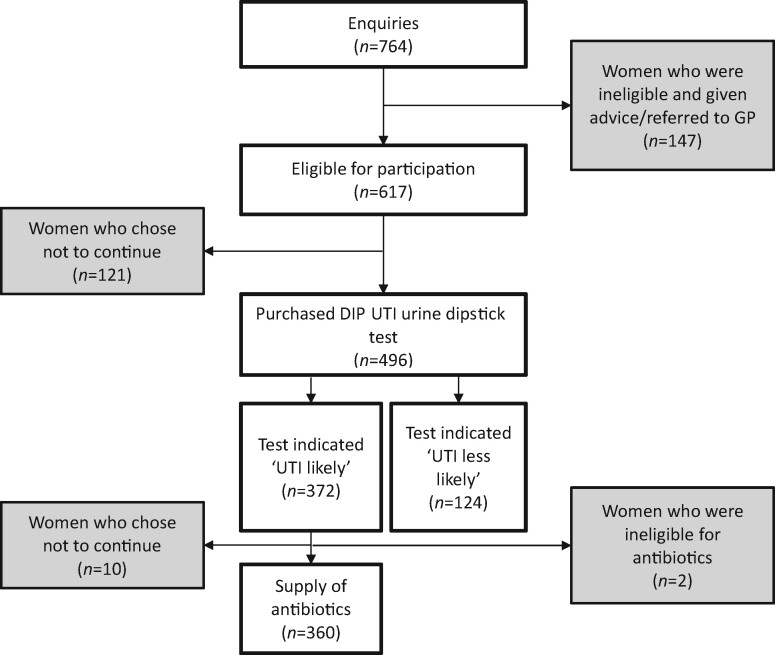
Flow of women through stages of the service. Shaded boxes represent patients excluded or who withdrew from the service.

Of the 764 women that enquired about the service, 617 (80.8%) were eligible to participate (Figure 2). At this stage, 147/764 (19.2%) women were ineligible to participate, reasons being: two or more episodes of UTI in the last 6 months or three or more in the last 12 months (62/764; 8.1%); symptoms lasting >7 days (45/764; 5.9%); currently taking antibiotics or previous use of antibiotics for UTI within the last 4 weeks (24/764; 3.1%); or pregnancy (3/764; 0.4%). Of those eligible to participate in the service, 496/617 (80.4%) chose to purchase a test while 121/617 (19.6%) chose not to continue with the service.

Urine dipstick test results indicated that 372/496 (75.0%) of women were likely to have a lower UTI and were offered antibiotic treatment. Of these, 360/372 (96.8%) women chose to purchase antibiotics supplied by use of a PGD. Two women were excluded at this stage due to ineligibility within the PGD criteria; the remaining 10 women chose not to continue due to reasons such as choosing to watch and wait, or wishing to speak to their GP owing to other complications. The majority of those that received treatment within the pharmacy were supplied nitrofurantoin (352/360; 97.8%) and where this was clinically unsuitable, trimethoprim was supplied for eight women.

**Figure 3.**
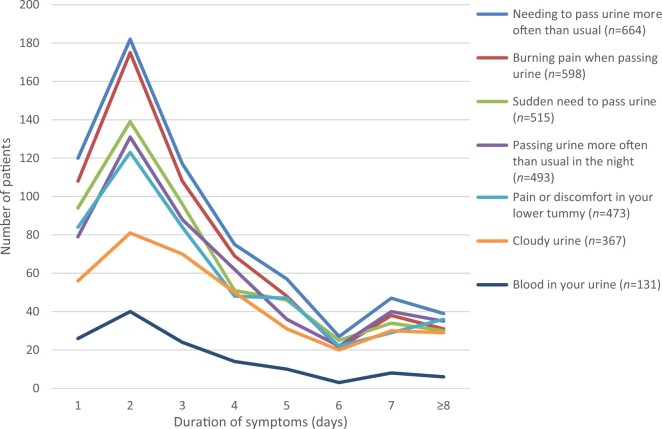
Frequency and duration of urinary symptoms (*n *=* *763).

Data on symptom type were captured for 763/764 women (Figure 3 shows symptom type by duration of symptoms) and individuals could select one or more from a list of seven symptoms. Over three-quarters of women reported needing to pass urine more often than usual (677/763; 88.7%), burning pain when passing urine (612/763; 80.2%), sudden need to pass urine (525/763; 68.8%), passing urine more often than usual at night (503/763; 65.9%) and pain or discomfort in lower tummy (482/763; 63.2%). The mean number of symptoms per individual was 4.3 (median 4.0, range 1 to 7). On average, those women for whom the results of the test indicated that a UTI was likely (*n *=* *372) had slightly more symptoms (mean 4.5, median 5) compared with those for whom the test indicated UTI was less likely (mean 3.8, median 4, *n *=* *124). The mean duration of symptoms for women (*n *=* *743) was 3.9 days (median 3 and mode 2). The data were positively skewed owing to the number of women having symptoms for longer than 7 days (full range 1 day to 3 months). Once all women with symptoms for over 7 days (*n *=* *45) were excluded, the mean duration of symptoms for women with ‘UTI less likely’ and ‘UTI likely’ test results was the same (3.0 days). There was little correlation between positivity rate and duration of symptoms (*R*^2^* *=* *0.0319).

National guidance for the diagnosis of UTI in women aged <65 years recommends women with two or more of the PHE three key symptoms are likely to have a lower UTI that requires antibiotic treatment.^6^ The guidance does not recommend use of urine dipstick testing for these women, but does recommend testing in women who only report one of the three symptoms. However, all women with urinary symptoms participating in this pilot service were required to use urine dipstick testing. Of those women who met the PHE three key symptoms for ‘UTI likely’ (Table 1), 281/350 (80.3%) with two or three of these symptoms had a ‘UTI likely’ test outcome, 83/123 (67.5%) women with one of the key symptoms tested ‘UTI likely’ and 8/22 (36.4%) women with none of the key symptoms tested ‘UTI likely’ (although they did have other symptoms recorded).

**Table 1.** Test outcome based on PHE three key symptoms: dysuria, new nocturia and cloudy urine (data available *n *=* *495)

	Number of the PHE three key symptoms		
	two or three (*n *=* *350)	one (*n *=* *123)	zero (*n *=* *22)
Test outcome ‘UTI likely’	281 (80.3%)	83 (67.5%)	8 (36.4%)
Test outcome ‘UTI less likely’	69 (19.7%)	40 (32.5%)	14 (63.6%)

The majority of women who accessed all elements of the service did so on the same day (331/360; 91.9%). For those women not completing all elements of the service on the same day, they returned the following day (22/360; 6.1%) or within 3 days (7/360, 1.9%).

Data indicating what action they would have taken if the service had not been available were reported for 301/360 (83.6%) women accessing both the urine dipstick test and subsequent treatment. The majority of these women (214/301; 71.1%) would have gone to their GP; over one-third would have attempted self-treatment as well or instead (116/301; 38.5%). Data on women who accessed the test only were less representative, as data were only available for 25.7% of women (35/136). Of these, 62.9% (22/35) would have accessed their GP and 40.0% (14/35) would have self-treated. Data were captured for five women who did not progress beyond the initial screening protocol; four of these would have accessed their GP and one would have self-treated.

## Discussion

The service included use of the PHE UTI guidance to clinically assess women before confirming eligibility to purchase a dipstick test. By doing this, pharmacy staff were able to triage women and give the most appropriate support, depending on the type and severity of symptoms.

Of those that were eligible to purchase the test, one-fifth chose not to do so. Women may have used the opportunity for discussion with the pharmacy team to understand whether there was a need to make a GP appointment (i.e. a ‘UTI likely’ test result) or accessed local NHS services where they could get free treatment. This was particularly the case in Sheffield, where 8/33 (24.2%) women chose not to proceed with the community pharmacy service (versus 113/584; 19.3% in London), possibly due to ease of access to the local NHS walk-in centre, which was opposite the pharmacy participating in the service. Pharmacists were encouraged to liaise with local GPs and service providers, as part of service training, to understand referral pathways and guidelines so that they could signpost appropriately.

For the purpose of the pilot, pharmacists used the PHE UTI guidance to identify women who were not suitable for the service. All women who were likely to have a lower UTI were required to use a urine dipstick test and treatment decisions were made on the outcome of the urine test result. This resulted in 69/350 (19.7%) women presenting with two or three of the PHE three key symptoms not being treated with antibiotics (instead provided with safety-netting advice), while eight women with none of the PHE three key symptoms were treated with antibiotics. These women presented with other clinical symptoms; however, these findings reinforce the importance of treating the individual based on the outcome of the assessment of clinical signs and symptoms and not on the test alone. Certain symptoms such as cloudy urine may be more difficult for women to notice (reported the least frequently of the PHE three key symptoms in our data) and they may place less importance on this compared with some of the more symptomatic issues of frequent and painful urination.

Six percent of women (45/764) were excluded owing to having had symptoms for longer than 7 days, indicating that many women delay accessing services, increasing the risk of bacteraemia.^10^ The majority of women suffered from multiple symptoms, with the rate of the test result being ‘UTI likely’ increasing as the number of symptoms increased.

The service was accessed by women of all ages (between 16 and 64 years) and levels of deprivation (based on the Carstairs index) despite having to pay for the service directly. The potential barrier of women having to visit the pharmacy on multiple occasions (for initial assessment and to purchase the test, and then again for the pharmacist to interpret the results and provide treatment if appropriate) did not appear to be an issue. The majority of women accessing all elements of the service did so on the same day.

Data on what women would have done if the service had not been available were mainly based on those that had completed all elements of the service pathway, due to the point at which this question was asked in the service pathway. The majority of these women would have gone to their GP; therefore, this service would be a direct substitution and cost saving if funded by the NHS.^27^^,^^28^ In addition, it would be more convenient as women would be able to access the service without an appointment (potentially saving time off work). Whilst data on those women who only accessed testing are limited, almost two-thirds would have seen their GP if the service had not been available, which would have been unnecessary given that a UTI was less likely (therefore cost saving). As pharmacists working within the community become more integrated into primary care networks, there is an opportunity for them to embed services such as this to support self-care and appropriate use of NHS resources. This would support changing behaviour of patients with UTIs to utilize community pharmacies as the first port of call (i.e. changing to a ‘pharmacy first’ mentality) and allow for effective triage and treatment within this environment. Whilst national recommendations exist on first- and second-line treatments for UTI, there are examples of local variations due to issues with resistance. Pharmacists need to engage with local networks so that they can work with other healthcare professionals to understand any variations in clinical pathways or treatment options, but also to work collaboratively in supporting the wider antimicrobial stewardship agenda. There is an opportunity for pharmacy staff within the community to help support patients with UTIs safely and effectively, in line with clinical guidelines and treatment pathways, regardless of whether this is funded by the NHS or the patients themselves, as long as patients are triaged appropriately and safety-netting advice is provided.

### Limitations

The data presented are based on a private pay service and whilst the clinical data are reflective of these women, it may not represent those that may have presented if the service was available free of charge on the NHS. Data were more representative of women accessing all elements of the service, compared with those accessing the test alone, which gives less certainty in reporting activity for this patient group. Pharmacists utilized the PHE UTI guidance as part of the service delivery as fully as they could, but were unable to send urine samples for culture. There were also no data available on patient follow-up (e.g. recurrent symptoms, treatment failure rates, hospital admissions); therefore, data on what patients would have done if the service were not available may not be truly representative of their opinion after resolution of their infection. It would be useful to include this in future service evaluations.

### Conclusions

Pharmacy teams were able to support females aged 16–64 years with uncomplicated lower UTI within the community pharmacy. Women were provided with self-care advice, treatment (if necessary) or triaged to a more appropriate service. The service helped to support the appropriate use of antibiotics and reduced demand on other NHS resources such as GP surgeries and urgent care settings. The recommendations to base diagnosis on clinical symptoms guided by appropriate use of urine dipstick testing (not just based on the test result alone) have already been incorporated in future service design.

## Supplementary Material

dlaa010_Supplementary_Data
